# Supplementary material for: Gon4l regulates notochord boundary formation and cell polarity underlying axis extension by repressing adhesion genes
Source: Nat Commun. 2018 Apr 3;9:1319. doi: 10.1038/s41467-018-03715-w (PMC5882663; doi:10.1038/s41467-018-03715-w)
Supplement: Supplementary file 1 — Supplementary Information(PDF 22557 kb) [file 41467_2018_3715_MOESM1_ESM.pdf]

SUPPLEMENTARY INFORMATION

Gon4l regulates notochord boundary formation and cell polarity underlying axis extension by repressing adhesion genes

Williams, *et al*.

SUPPLEMENTARY FIGURES

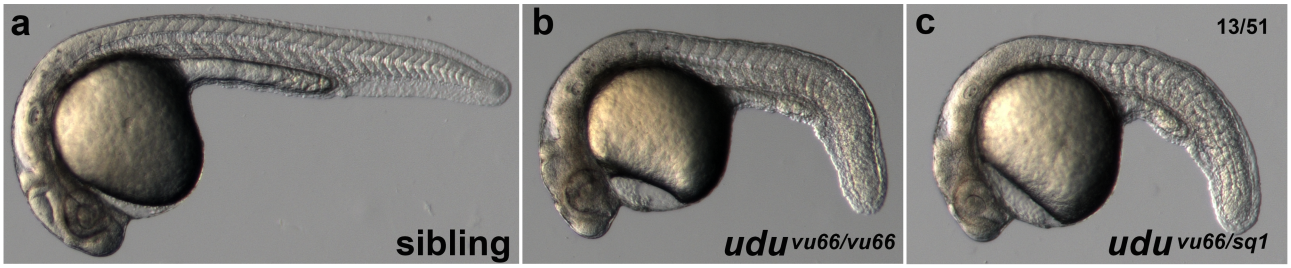

Supplementary Figure 1: *vu66* is a new allele of *ugly duckling*
Live day 1 WT (a) and *udu*-/- (b,c) embryos. **c**) Representative embryo from a complementation cross between a *vu66* heterozygote and an udu^{sq1} heterozygote. Phenotype of *vu66*/*sq1* mutants (c) resembles *vu66/vu66* (b), indicating failure to complement. Fraction indicates the number of embryos with the pictured genotype/phenotype over total embryos in the clutch.

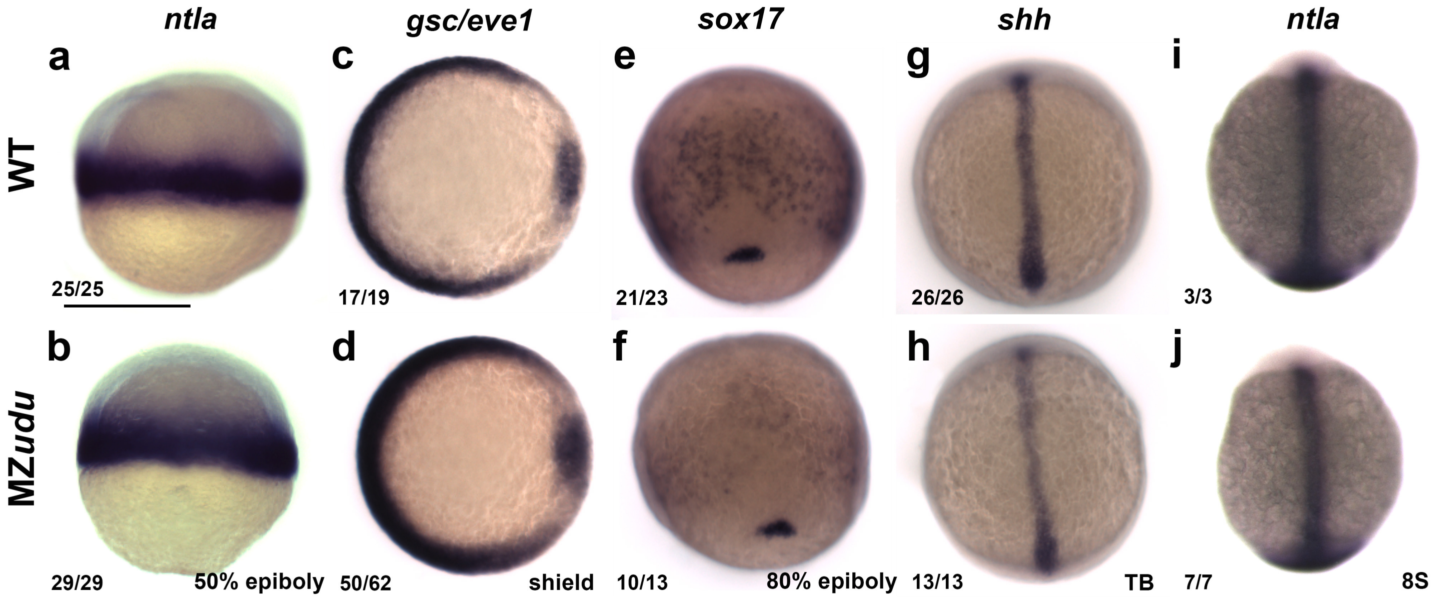

Supplementary Figure 2: Maternal zygotic (MZ)*udu-/-* embryos specify all three germ layers
Whole mount *in situ* hybridizations (WISH) in WT (top row) and MZ*udu-/-* embryos (bottom row) at the stages indicated. **a-b**) *ntla* marks nascent mesoderm. **c-d**) *gsc* and *eve1* mark the embryonic shield and ventral mesoderm, respectively. **e-f**) *sox17* marks endoderm and dorsal forerunner cells. **g-j**) *shh* (g-h) and *ntla* (i-j) mark axial mesoderm. Fractions indicate the number of embryos with the pictured phenotype over the number of embryos examined. Images depict a dorsal view with anterior/animal pole up in all images except c-d in which dorsal is to the right. Scale bar is 300μm.

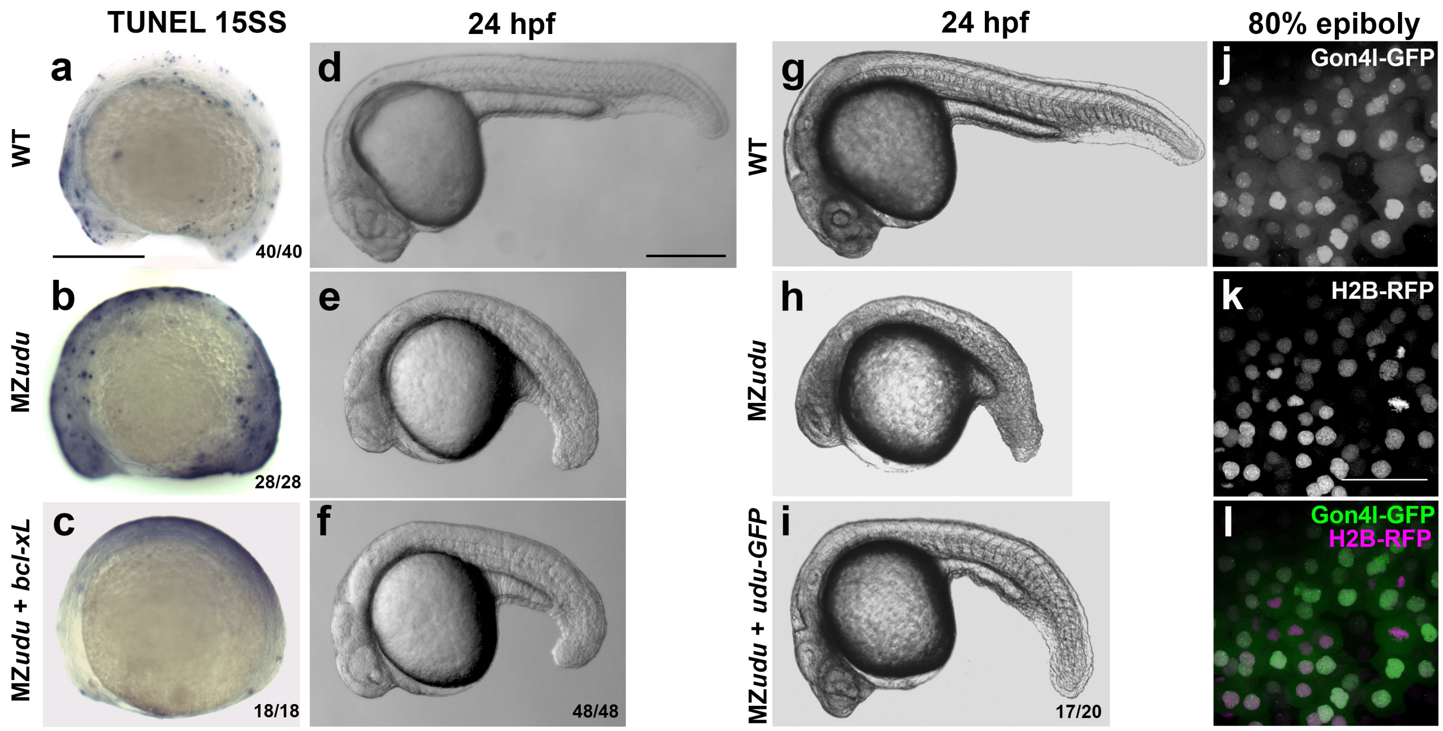

Supplementary Figure 3: Increased apoptosis in MZ*udu-/-* embryos is not causative of axis extension defects

a-c) TUNEL staining in WT (a), MZ*udu-/-* (b), and MZ*udu* + *bcl-xL* RNA (c) at 15 somite stage. **d-i**) Live WT (d,g), MZ*udu-/-* (e,h), MZ*udu* + *bcl-xL* (f), and MZ*udu* + *25pg udu-gfp* (i) embryos at 24hpf. Fractions indicate the number of embryos with the pictured phenotype over the number of embryos examined. **j-l**) Live WT embryos at 80% epiboly expressing Gon4l-GFP (j) and Histone2B-RFP (k), merged channels in l. Images are representative of four independent trials. Scale bars are 300μm in a-i, 50μm in k. Anterior is to the left.

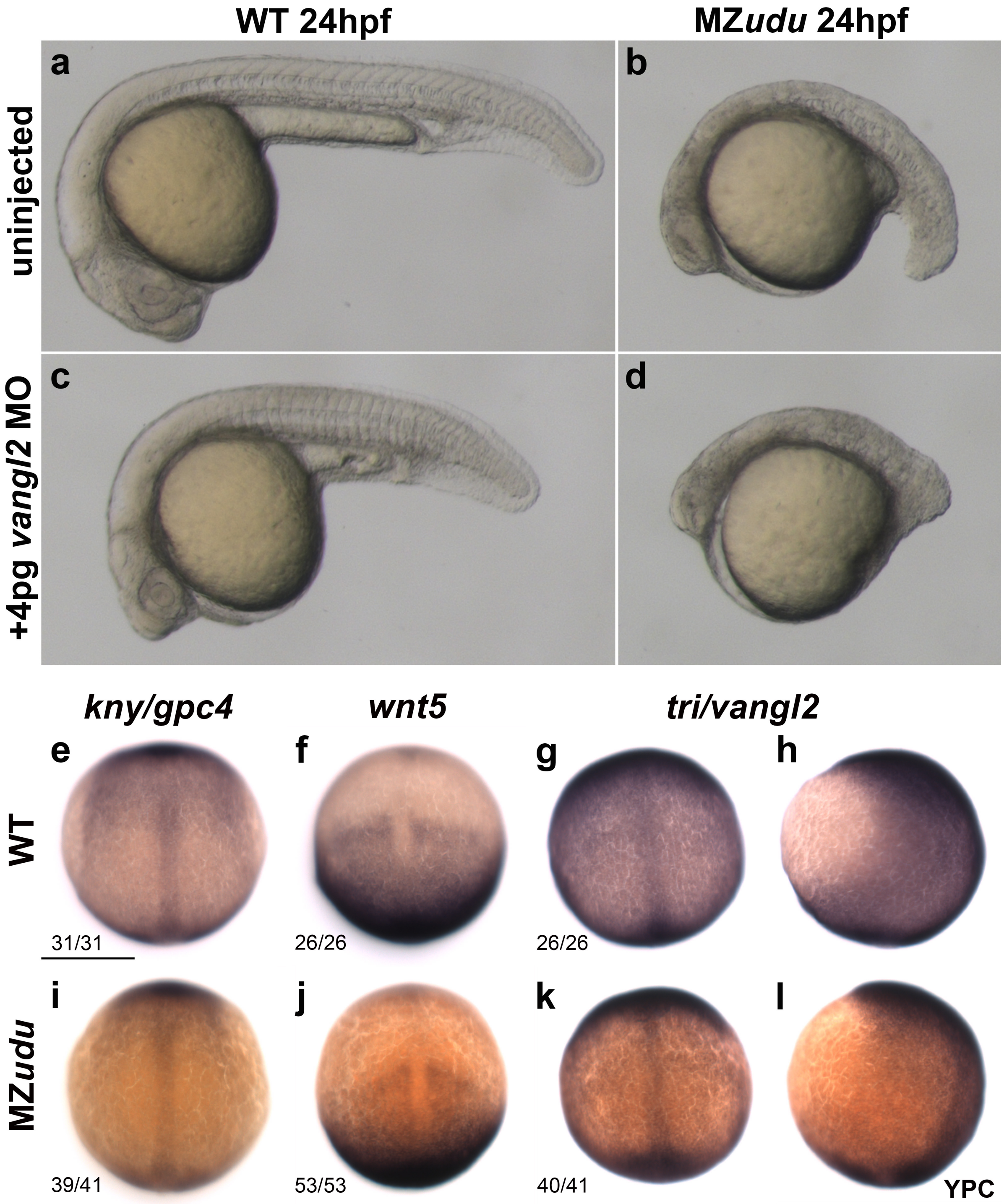

Supplementary Figure 4: Gon4l functions in parallel to PCP signaling
a-b) Live uninjected WT (a) and MZ*udu-/-* (b) embryos at 24hpf. **c-d**) Live 24hpf WT (c) and MZ*udu-/-* (d) embryos injected with 4pg MO1-*vangl2* morpholino. Images are representative of two independent trials. **e-l**) WISH for the PCP genes *kny/gpc4* (e,i), *wnt5* (f,j), and *tri/vangl2* (g-h, k-l) in WT (top row) and MZ*udu-/-* (bottom row) embryos at YPC stage. Fractions indicate the number of embryos with the pictured phenotype over the number of embryos examined. Anterior is up, dorsal is to the right in h,l. Scale bar is 300 μm.

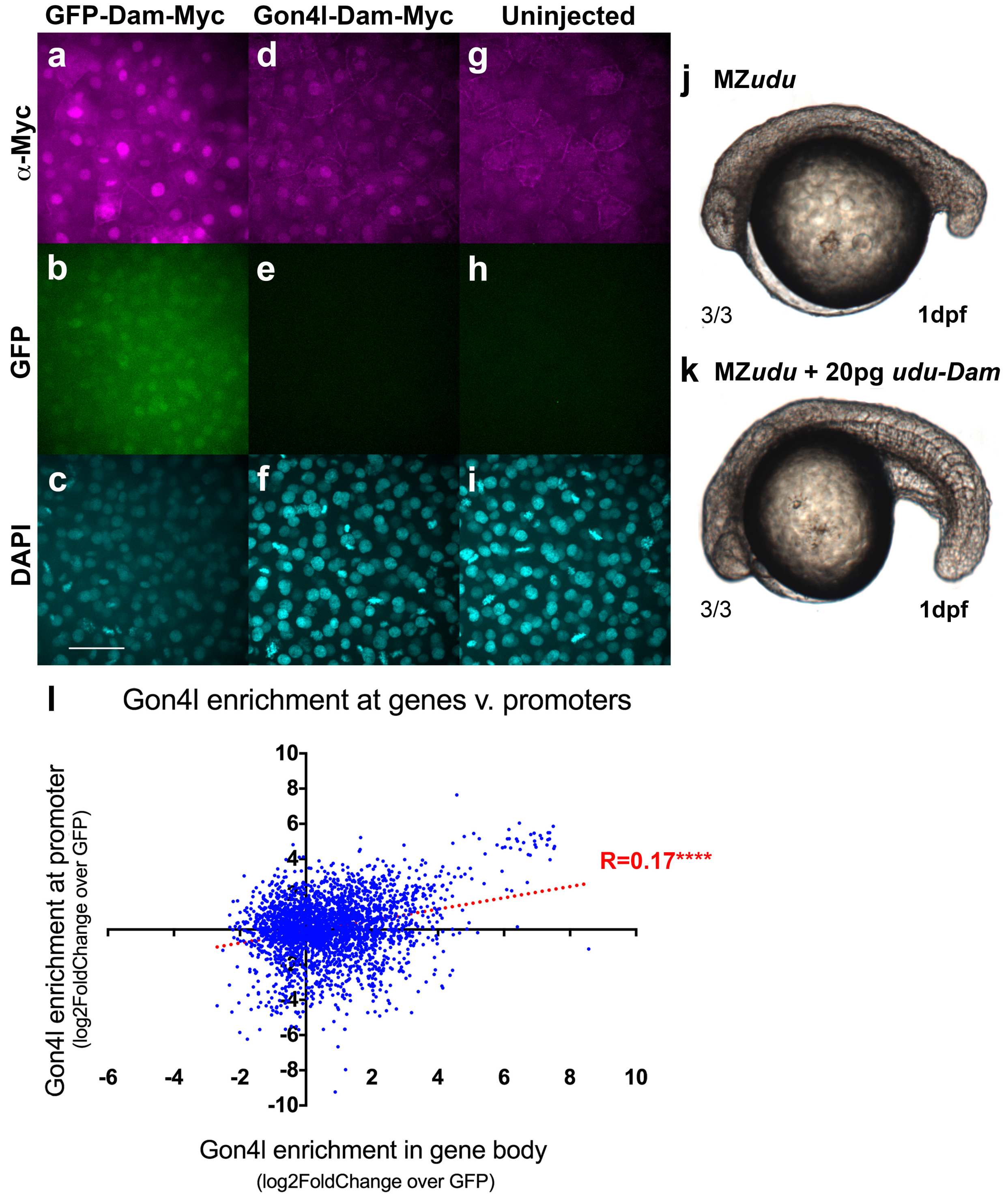

Supplementary Figure 5: Dam fusion proteins are functional

a-i) Anti-Myc Immunofluorescent staining for Dam-Myc fusion proteins in WT embryos injected with *gfp-dam-myc* (a-c) or *udu-dam-myc* (d-f) RNA or uninjected controls (g-i) at blastula stage. Images are representative of multiple embryos from a single trial. **j**) Uninjected MZ*udu-/-* sibling at 1 dpf. **k**) MZ*udu-/-* injected with 20pg *udu-dam* RNA. Fractions indicate number of embryos with the pictured phenotype over the number of embryos examined. **l**) Correlation between Gon4l enrichment across promoters and enrichment across gene bodies of genes with regions of significant Gon4l association (Spearmann correlation ****p<0.0001). Dotted line is linear regression of correlation. Negative values indicate depletion of Gon4l relative to GFP controls. Scale bar is 50μm.

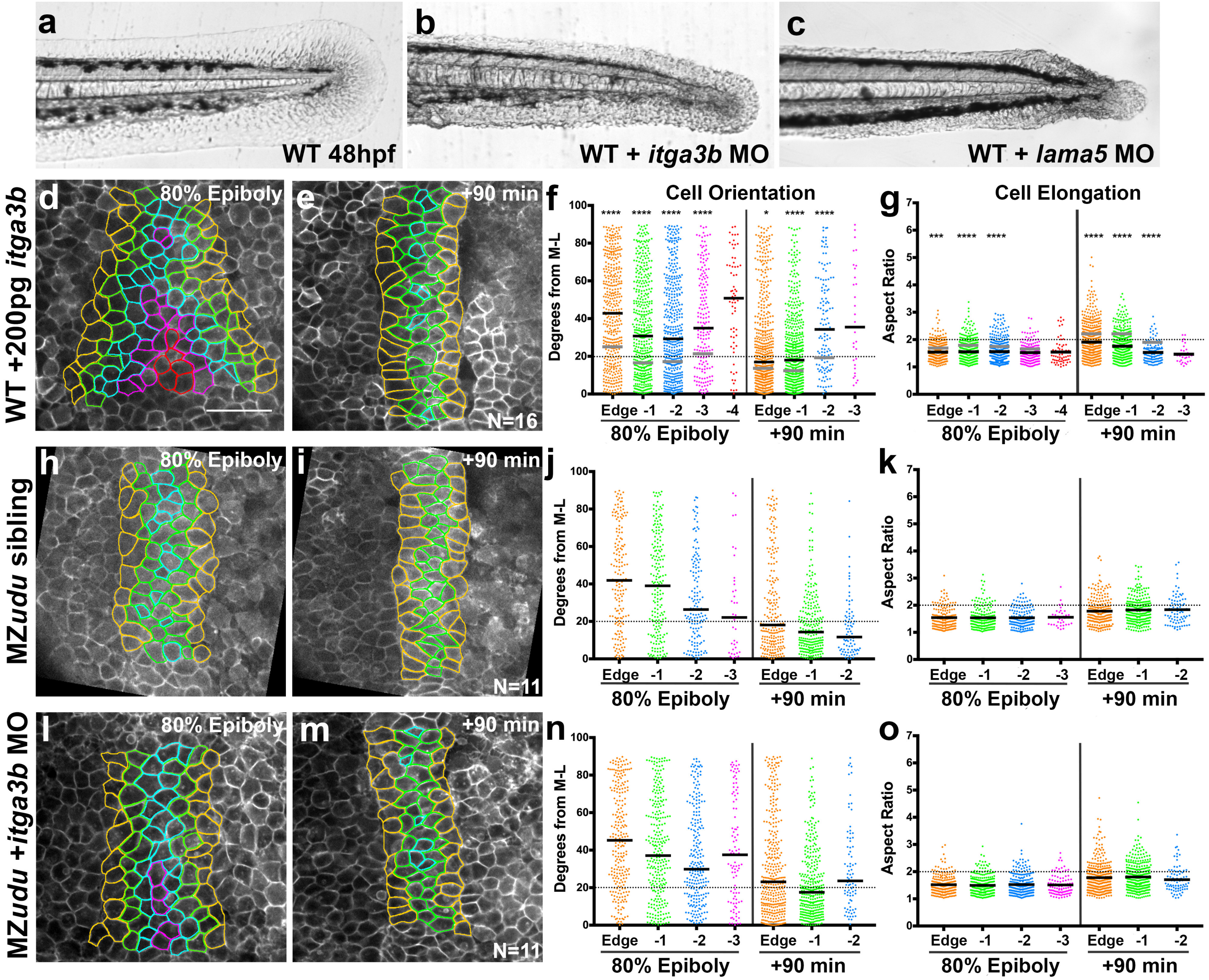

Supplementary Figure 6: Reduction of Integrinα3b does not suppress cell polarity defects in MZ*udu*-/- gastrulae

a-c) Live images of caudal fins of an uninjected WT embryo (a) and those injected with 2ng *itga3b* MO (b) or 1ng *lama5* MO (c) at 48hpf (note recapitulation of mutant phenotypes[1]). Images are representative of all MO-injected embryos from three (*itga3b*) or four (*lama5*) independent trials. **d-g**) Quantification of axial mesoderm cell orientation (f) and elongation (g) in *itga3b* overexpressing WT gastrulae. Asterisks indicate significant differences compared to WT controls (Kolmogorov-Smirnov (f) and Mann-Whitney (g) tests, *p<0.05, ***p<0.001, ****p<0.0001). Black bars are median values in f, mean values in g; medians and means of WT are shown as gray bars in f-g. **h-o**) Quantification of axial mesoderm cell orientation and elongation in control MZ*udu*-/- siblings (h-k) and MZ*udu-/-* injected with 2ng *itga3b* MO (l-o) at the time points indicated. Graphs and color coding as in Fig.3. Bars are median values in j, n; bars are mean values in k, o. N indicates the number of embryos analyzed, scale bar is 50μm.

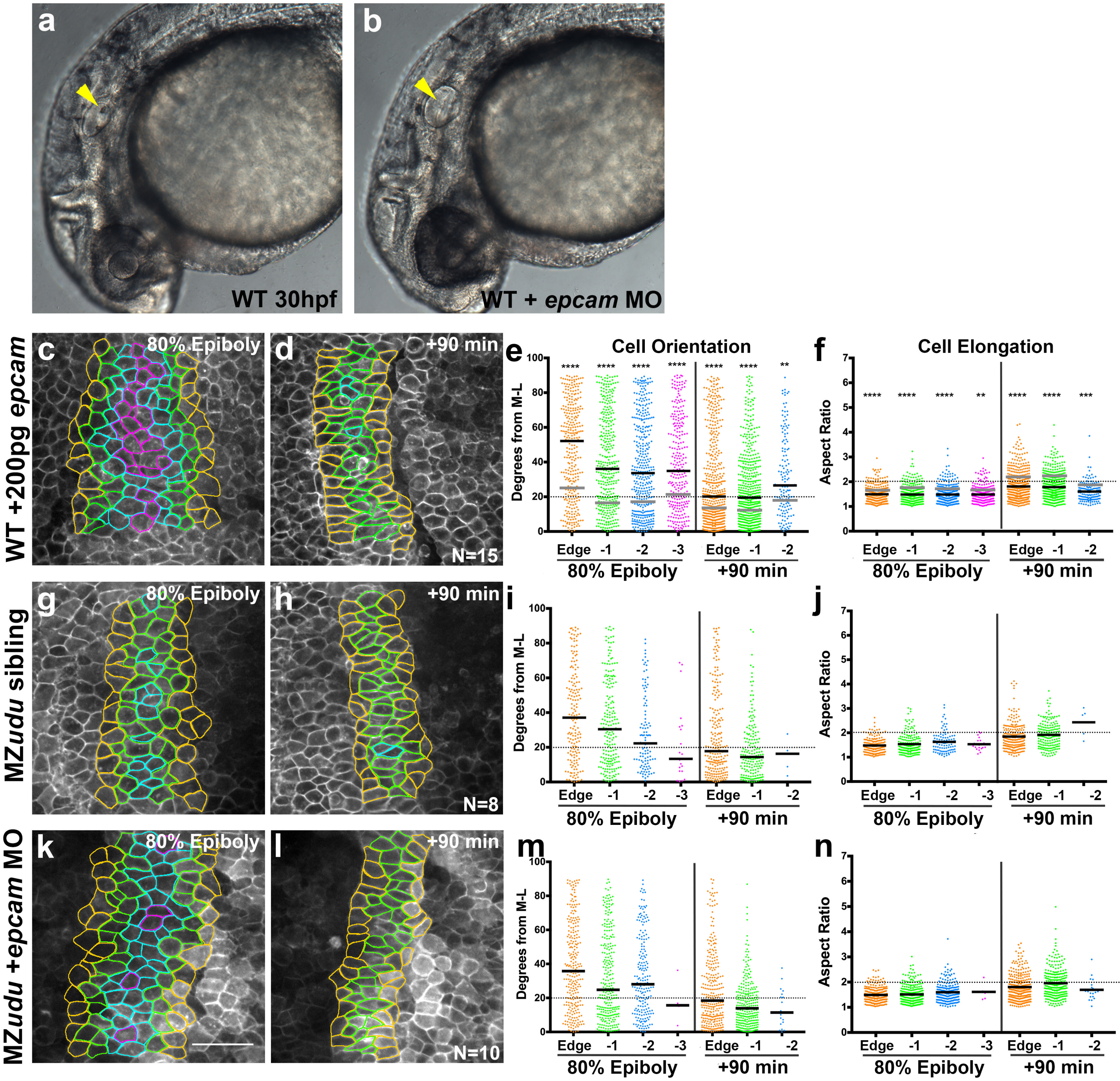

Supplementary Figure 7: Reduction of EpCAM does not suppress cell polarity defects in MZ*udu*-/- gastrulae

a-b) Live images of otic vesicles in WT embryos at 30hpf either (a) uninjected or (b) injected with 1ng *epcam* MO. Arrowheads mark otoliths (or lack thereof). Approximately 36% of *epcam* MO-injected embryos from three independent trials lacked otoliths as shown in b, an additional 56% exhibited smaller otoliths (both phenotypes are observed in *epcam* mutants[2]). **c-f**) Quantification of axial mesoderm cell orientation (e) and elongation (f) in *epcam* overexpressing WT gastrulae. Asterisks indicate significant differences compared to WT controls (Kolmogorov-Smirnov (e) and Mann-Whitney (f) tests, **p<0.01, ***p<0.001, ****p<0.0001). Black bars are median values in e, mean values in f; medians and means of WT are shown as gray bars in e-f. **g-n**) Quantification of axial mesoderm cell orientation and elongation in control MZ*udu*-/- siblings (g-j) and MZ*udu*-/- injected with 1ng *epcam* MO (k-n) at the time points indicated. Graphs and color coding as in Fig. 3. Bars are median values in i, m; bars are mean values in j, n. N indicates the number of embryos analyzed, scale bar is 50μm.

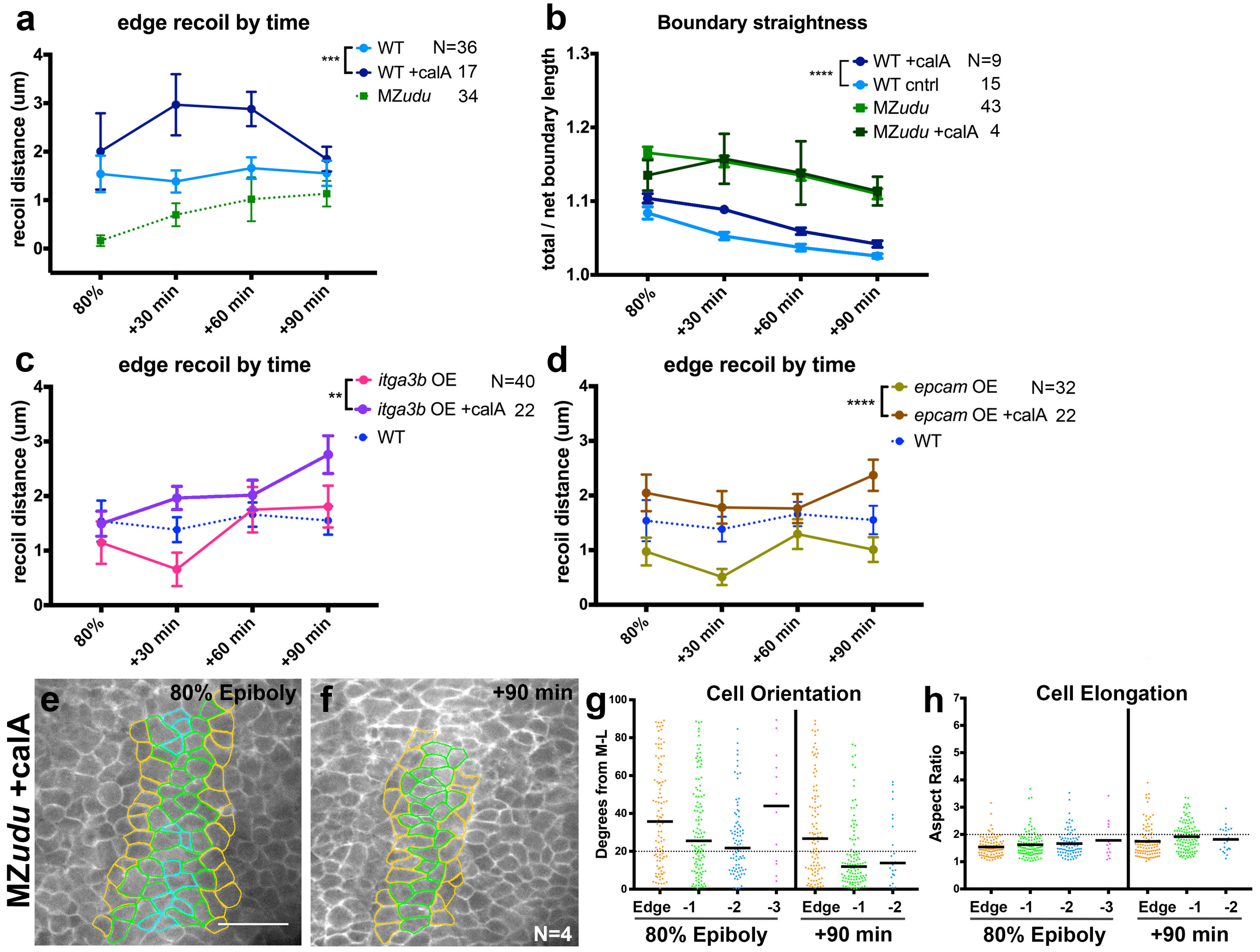

Supplementary Figure 8: Calyculin A treatment increases notochord boundary tension without improving MZ*udu*-/- boundary straightness or cell polarity

a) Quantification of cell vertex recoil distance upon laser ablation of Edge cell interfaces at the time points indicated in MZ*udu-/-*, WT, and Calyculin A-treated WT gastrulae. Symbols are means with SEM (2-way ANOVA, ***p=0.0006). **b**) Quantification of boundary straightness in WT and MZ*udu-/-* gastrulae with or without Calyculin A treatment. Symbols are means with SEM (2-way ANOVA, ****p<0.0001). **c-d**) Cell vertex recoil distance upon laser ablation of Edge cell interfaces at the time points indicated in WT embryos overexpressing *itga3b* (c) or *epcam* (d) with or without Calyculin A treatment. Symbols are means with SEM (2-way ANOVA, **p=0.027, ****p<0.0001). **e-h**) Quantification of axial mesoderm cell orientation (g) and elongation (h) in Calyculin A-treated MZ*udu-/-* gastrulae. Graphs and color coding as in Fig.3. Bars are median values in g and mean values in h. Scale bar is 50μm. N indicates the number of embryos analyzed.

SUPPLEMENTARY REFERENCES

1. Carney, T.J. *et al.* Genetic analysis of fin development in zebrafish identifies furin and hemicentin1 as potential novel fraser syndrome disease genes. *PLoS Genet* **6**, e1000907 (2010).
2. Slanchev, K. *et al.* The epithelial cell adhesion molecule EpCAM is required for epithelial morphogenesis and integrity during zebrafish epiboly and skin development. *PLoS Genet* **5**, e1000563 (2009).
