# Supplementary material for: Gon4l regulates notochord boundary formation and cell polarity underlying axis extension by repressing adhesion genes
Source: Nat Commun. 2018 Apr 3;9:1319. doi: 10.1038/s41467-018-03715-w (PMC5882663; doi:10.1038/s41467-018-03715-w)
Supplement: Supplementary file 3 — Description of Additional Supplementary Files(DOCX 15 kb) [file 41467_2018_3715_MOESM3_ESM.docx]

**Description of Additional Supplementary Files**

File Name: Supplementary Data 1

Description: Genes differentially expressed in MZ*udu* mutants v. WTs List of genes identified by RNA-seq as differentially expressed between WT and MZ*udu-/-* embryos at tailbud stage. Cutoff for differential expression was adjusted p-value ≤0.01, log2 fold change ≥1.

File Name: Supplementary Data 2

Description: Gon4l-enriched gene bodies List of genes containing at least one region within the gene body (transcription start site (TSS) to transcription end site) of Gon4l enrichment over GFP controls with adjusted p-value ≤0.01, log2 fold change ≥2. Values included are log2 fold change Gon4l/GFP across the entire gene body.

File Name: Supplementary Data 3

Description: Gon4l-enriched promoters List of genes whose promoters (2kb upstream of TSS) contain at least one region of Gon4l enrichment over GFP controls with adjusted p-value ≤0.01, log2 fold change ≥2. Values included are log2 fold change Gon4l/GFP across the entire promoter.
